# Highly Efficient Biotransformation of Notoginsenoside R1 into Ginsenoside Rg1 by *Dictyoglomus thermophilum* *β*-xylosidase Xln-DT

**DOI:** 10.4014/jmb.2111.11020

**Published:** 2022-02-10

**Authors:** Qi Li, Lei Wang, Xianying Fang, Linguo Zhao

**Affiliations:** 1 Co-innovation Center for Sustainable Forestry in Southern China, Nanjing Forestry University, 159 Long Pan Road, Nanjing 210037, P.R. China; 2 College of Chemical Engineering, Nanjing Forestry University, 159 Long Pan Road, Nanjing 210037, P.R. China; 3 Jiangsu Co-Innovation Center of Efficient Processing and Utilization of Forest Resources, Nanjing Forestry University, Nanjing 210037, P.R. China

**Keywords:** Ginsenoside Rg1, β-xylosidase, biotransformation, anti-fatigue activity

## Abstract

Notoginsenoside R1 and ginsenoside Rg1 are the main active ingredients of *Panax notoginseng*, exhibiting anti-fatigue, anti-tumor, anti-inflammatory, and other activities. In a previous study, a GH39 β-xylosidase Xln-DT was responsible for the bioconversion of saponin, a natural active substance with a xylose group, with high selectivity for cleaving the outer xylose moiety of notoginsenoside R1 at the C-6 position, producing ginsenoside Rg1 with potent anti-fatigue activity. The optimal bioconversion temperature, pH, and enzyme dosage were obtained by optimizing the transformation conditions. Under optimal conditions (pH 6.0, 75°C, enzyme dosage 1.0 U/ml), 1.0 g/l of notoginsenoside R1 was converted into 0.86 g/l of ginsenoside Rg1 within 30 min, with a molar conversion rate of approximately 100%. Furthermore, the in vivo anti-fatigue activity of notoginsenoside R1 and ginsenoside Rg1 were compared using a suitable rat model. Compared with the control group, the forced swimming time to exhaustion was prolonged in mice by 17.3% in the Rg1 high group (20 mg/kg·d). Additionally, the levels of hepatic glycogen (69.9-83.3% increase) and muscle glycogen (36.9-93.6% increase) were increased. In the Rg1 group, hemoglobin levels were also distinctly increased by treatment concentrations. Our findings indicate that treatment with ginsenoside Rg1 enhances the anti-fatigue effects. In this study, we reveal a GH39 β-xylosidase displaying excellent hydrolytic activity to produce ginsenoside Rg1 in the pharmaceutical and food industries.

## Introduction

Fatigue is a subjective feeling of extreme tiredness manifested after physical activity, demonstrating the predominant symptoms of drowsiness, dyspnea, and weakness after exercise [1, 2]. Occasionally, almost everyone experiences tiredness in daily life. This kind of temporary fatigue usually has a clear rationale and can be quickly relieved by adequate resting. However, long-term or accumulated fatigue greatly affects an individual's health, leads to several diseases (malnutrition, diabetes, hyperthyroidism, and malignant tumor disease), and can even result in death owing to overwork [3, 4]. In China, from ancient times to date, Chinese herbal tonics such as ginseng, *Polygonatum*, *Astragalus*, and *Ganoderma lucidum* have been commonly used for as anti-fatigue agents [5-7].

Ginseng saponins (Ginsenosides) are the major bioactive metabolites in the plants of the genus *Panax* (Family Araliaceae), specifically *Panax ginseng* C. A. Meyer, *Panax notoginseng*, and *Panax quinquefolius* [8-10]. Ginsenosides have been regarded as remarkable ingredients possessing pharmacological activities, including anti-fatigue, anti-inflammatory, anti-tumor, and anti-allergic activities [11-13]. Ginsenoside Rg1 (Rg1), one of the major active ginsenosides present in *Panax ginseng* in traditional Chinese medicine, has demonstrated therapeutic efficacy in Alzheimer's disease, neurodegenerative diseases, aging, fatigue, cardiovascular diseases, as well as cancer prevention [14-16]. Therefore, the Chinese Pharmacopoeia (Edition 2015) has listed Rg1 as a major index component for the quality control of ginseng herbs [17]. However, it remains considerably difficult to improve the purity of the precise Rg1 quantity in ginseng herbs owing to the presence of other homologous components, presenting markedly similar physicochemical properties as the target compound. Notoginsenoside R1 (R1), the main component of *Panax notoginseng*, has an additional xylose group at the C-6 position when compared with Rg1; however, raw materials for *Panax notoginseng* are substantially cheaper, with a higher saponin content. Therefore, if the outer xylose moieties at the C-6 carbon of R1 can be effectively removed for conversion into the homologous component, the major active target compound Rg1, the purity of Rg1 can be greatly improved.

For ginsenoside conversion, methods such as acid hydrolysis, heat treatment, steam treatment, as well as microbial and enzymatic transformation have been previously utilized [18-20]. Of the above-listed methods, enzymatic transformation has demonstrated potential owing to its mild reaction conditions and high specificity. However, the determination of a highly thermophilic recombinant enzyme with the ability to convert R1 to Rg1 remains challenging. Thermophilic β-xylosidase has a natural advantage and can improve the solubility during substrate transformation. However, to date, only Shin and Zhang have reported that GH39 β-xylosidases from *Thermoanaerobacterium thermosaccharolyticum* and *Sphingomonas* sp. JB13, respectively, can hydrolyze R1 [21, 22].

β-Xylosidase, a major component of hemicellulose degrading enzymes, is well known to degrade the non-reducing ends of β-1,4-linked D-xylose residues to release xylose and has been investigated in a variety of microorganisms, including bacteria, archaea, fungi, and plants [23-25]. In addition to the degradation of xylooligosaccharides, β-xylosidase has great potential in biotechnological applications, especially in the food, medicine, pulp, and paper industries [26-28]. Although numerous β-xylosidases from various sources have been cloned, purified, and biochemically characterized, data regarding highly thermostable β-xylosidases from the GH39 family, demonstrating high specificity for transforming natural active substances, remain scarce. Currently, there is growing interest in thermos-tolerant and highly xylose resistant β-xylosidases for industrial processes, as high temperatures are required to increase the solubility of substrates and reduce the risk of contamination; moreover, high xylose tolerance prevents the feedback inhibition of xylose in the reaction system. Hence, the search for thermostable and xylose-tolerant β-xylosidases, with potential application value in ginsenoside bioconversion, has gained momentum in recent times.

Previously, we cloned and characterized a thermostable and xylose-tolerant GH39 β-xylosidase Xln-DT from a thermophilic bacterium, *Dictyoglomus thermophilum*, which has demonstrated a high selectivity for cleaving the outer xylose group of natural saponins [29]. In the present study, we optimized the biotransformation conditions of R1 by β-xylosidase Xln-DT. Furthermore, we compared the anti-fatigue activity of R1 and Rg1 in a rat model. Our results showed that β-xylosidase Xln-DT demonstrated high selectivity for xylose hydrolysis in R1, whereas the hydrolysate Rg1 could improve the anti-fatigue activity in vivo. These remarkable properties render Xln-DT more suitable for producing Rg1 in the pharmaceutical and food industries.

## Materials and Methods

### Bacterial Strains, Growth Media, and Materials

The recombinant strain Xln-DT-pet28a was constructed and preserved at Microbial Technology Research Laboratory, Nanjing Forestry University (NJFU). The purified recombinant β-xylosidases (Tth XyB3, Tpxy3, XlnD, and TthXyl) were prepared by Microbial Technology Research Laboratory and Jiangsu Key Laboratory for the Chemistry & Utilization of Agricultural and Forest Biomass, NJFU. Data regarding β-xylosidases used in the experiment are shown in Table 1. The determination of optimum temperature and pH for β-xylosidases was using *p*-nitrophenyl-β-D-xylopyranoside (*p*NPX) as the substrate.

The Lysogeny broth (LB) medium was prepared using yeast extract (5 g/l), peptone (10 g/l), and NaCl (10 g/l), followed by the addition kanamycin (100 mg/ml), and 15 g/l agar.

The substrate *p*NPX was purchased from Sigma-Aldrich (USA). Standard notoginsenoside R1 (> 98% Purity, high-pressure liquid chromatography (HPLC)) and ginsenoside Rg1 (> 98% Purity, HPLC) were purchased from Chengdu Must Bio-Technology (China). All other reagents used were of analytical purity.

### Protein Purification and Enzyme Assay

The recombinant strain Xln-DT-pet28a was transformed into *Escherichia coli* BL21 (DE3). For β-xylosidase Xln-DT expression, transformed cells were grown in LB medium containing 100 mg/ml of kanamycin at 37°C, followed by induction of recombinant Xln-DT expression by adding 0.01 mM isopropyl-β-D-thiogalactopyranoside (IPTG), demonstrating an optical density of 0.8 at 600 nm (OD_600_), and further incubation at 28°C overnight. The culture was harvested by centrifugation at 8, 000 ×*g* (4°C) for 10 min, washed with distilled water several times, and resuspended in 1×binding buffer (pH 7.9, 5 mM imidazole, 0.5 mM NaCl, and 20 mM Tris-HCl). After sonication, the cell extracts were heat-treated at 75°C for 30 min, then cooled in an ice-bath, and centrifuged at 8,000 ×*g* (4°C) for 30 min. Finally, the supernatants were loaded and purified on a 2 ml Ni^2+^-NTA affinity chromatography column (Novagen, USA), and the enzyme protein was collected by eluting with Tris-HCl buffer containing 100 mM imidazole. After dialysis and ultrafiltration, the proteins were examined by SDS-PAGE gel, and the protein bands were analyzed using an image analysis system (Bio-Rad, USA) [30]. The purified protein concentration was measured using the Bradford method using bovine serum albumin (BSA) as the standard.

For purified β-xylosidase, the specific activity assay was performed using *p*NPX as a substrate. Under standard assay conditions, the purified enzyme was incubated with 10 μl *p*NPX (10 mM) in 50 mM sodium phosphate buffer (pH 6.0) for 10 min at 75°C. The total volume of the assay system was 100 μl. The reaction was stopped after 10 min by adding 300 μl of 1 M Na_2_CO_3_ [23, 30]. The released *p*NP was immediately measured at 405 nm. One enzyme activity unit (1 U) is defined as the amount of enzyme necessary to liberate 1 μmol of *p*NP per min, under the assay conditions. For every sample, the activity was measured in triplicate.

### Enzymatic Transformation of Notoginsenoside R1

For R1 as the substrate, five purified β-xylosidases (Xln-DT, Tth xynB3, Tpxy3, TthXyl, and XlnD) were selected to hydrolyze R1, using a reaction mixture containing 50 mM sodium phosphate buffer (pH 6.0), 1 g/l R1 (DMSO as a solvent), and 1.0 U/ml of β-xylosidase, incubated for 30 min at 75°C, and then stopped by adding twice the volume of methanol. The methanol extract was assayed by HPLC. Using HPLC, the reaction was measured at various time points. Control samples were prepared using only the substrate without enzyme, as well as using the enzyme without substrate.

To select suitable bioconversion conditions, the main factors influencing the transformation of R1 by Xln-DT were investigated. The main factors and the variation ranges were as follows: pH (5.0, 5.5, 6.0, 6.5, 7.0, and 8.0), temperature (60, 65, 70, 75, 80, and 85°C), enzyme dosage (0.1, 0.3, 0.5, 1.0, 1.5, 2.0, and 3.0 U/ml). To confirm the influence of R1 on β-xylosidase Xln-DT, different concentrations of R1 (1.0, 2.0, 3.0, 5.0, and 10.0 g/l) were incubated with Xln-DT.

The molar conversion rate of R1 to Rg1, expressed as the bioconversion rate (%), was calculated using the following formula (as given by Eq. 1):

Bioconversion rate (%) = [*C_t_/M_t_]*/[*C_i_/M_i_*]×100% (1)

Where, Ci is the initial concentration of the substrate R1, Mi is the molar mass of the substrate R1, Ct is the product concentration of Rg1 after time t, and Mt is the molar mass of Rg1.

**Fig. 1:**
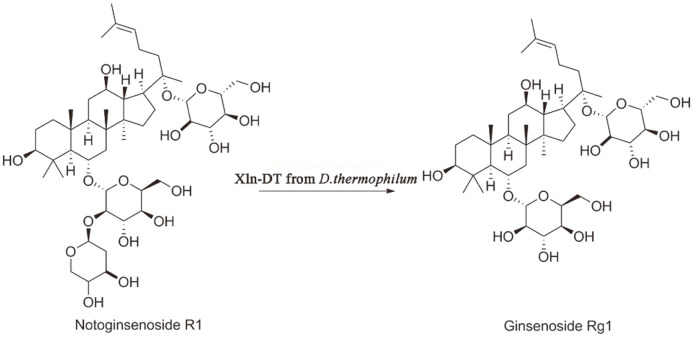
The chemical structures and biotransformation pathway of notoginsenoside R1.

The concentrations of the substrate R1 and product Rg1 were calculated according to the standard equations (y=2805.5x-35.267, R2=0.9986 for R1 and y=2902.2x+153.36, R2=0.9966 for Rg1). The structures and proposed biotransformation pathway are shown in Fig. 1.

### Compound Preparation

Notoginsenoside R1 (purity over 98%) was procured from a supplier. The hydrolysate of notoginsenoside R1 was prepared by microporous resin D101 (30 g of microporous resin D101 loading column, soaked overnight in ethanol, with pretreatment using 5% HCl and 2% NaOH).

After enzymatic transformation by β-xylosidase Xln-DT, the mixture mainly contained Rg1 and Xln-DT. The reaction mixture was prepared by centrifugation at 8,000 ×*g* for 10 min, at 25°C. The supernatant was steamed, dissolved in 10 ml distilled water. Xln-DT were inactivated by high temperature (100°C for 10 min), and the mixture was passed through the column twice for complete adsorption. Then, the adsorbed samples were washed twice with 150 ml of ultra-pure water and eluted with 80% (volume fraction) ethanol, and one bottle was collected every 100 ml. After eluting with 80% ethanol, the components containing Rg1 were evaporated, dissolved in 10 ml ultra-pure water, and freeze-dried.

The content of ginsenoside Rg1 in the samples was detected by HPLC until no Rg1 was eluted. The components containing Rg1 were evaporated, dissolved in 10 ml ultra-pure water (ultrasound-assisted dissolution), freeze-dried, then followed by preparation of the Rg1 powder.

### Assay of Ginsenoside Rg1 and Notoginsenoside R1 by HPLC and Liquid Chromatography-Mass Spectrometry (LC-MS)

R1 and Rg1 were analyzed using an HPLC 1260 system (Agilent, USA) and a C18 column (4.6 × 250 mm; internal diameter, 5 μm; S.No. USNH017518, USA), using distilled water (A) and acetonitrile (B) as the mobile phase, with a gradient elution of 20% (B) from 0 to 3 min, 20-47% (B) from 3 to 20 min, finally, using A/B ratios of 53:47 to 80:20 till run time of 22 min. The flow rate was 0.4 ml/min, the injection volume was 10 μL for each sample, and detection was performed by measuring absorbance at 203 nm. The hydrolysis product was identified by HPLC using standards, as well as LC-MS. All mass spectrometric experiments were performed on a triple-quadrupole tandem mass spectrometer (Agilent, USA). The column temperature was 300°C.

### Experimental Animals and Administration

In total, 60 male ICR mice (4-6 weeks old, weighing 26-28 g) were purchased from Qinglong Mountain Animal Reproduction Center (China), and were acclimatized for 5 days before experimentation. Food and water were provided ad libitum. All animal experimental procedures were performed in accordance with the internationally accepted Guide for Care and Use of Laboratory Animals (Ministry of Science and Technology of China, 2006), ethical regulations of Nanjing Forestry University and the study protocol was approved by the Animal Care and Protection Committee of Gulou Hospital, Nanjing University (SYXK 2004-0013). All efforts were made to minimize the suffering of animals, as well as to reduce the number of animals used.

Mice were weighed before experimentation and were randomly divided into 5 groups: Group 1 (control) was administered 1×PBS (phosphate-buffered saline) (pH 7.4); Group 2 and Group 3 were administered low- and high-dosage R1 (5 and 20 mg/kg, respectively); Group 4 and Group 5 were administered low- and high-dosage of Rg1 (5 and 20 mg/kg, respectively). Mice in the same group were kept individually in the same cage. For all experiments, each group was composed of 12 mice.

### Changes in Body Weight

Prior to testing, the two test samples, as well as two dose levels, were prepared, and administered by oral gavage once a day, for 2 weeks. Changes in mice body weight were observed during the initial, intermediate, and final stages of testing. Sixty ICR male mice were used for the following tests, performed in a randomized double-blind manner.

### Forced Swimming Test

After two weeks, the mice underwent a forced swimming test. The procedure used was similar to that previously described in the literature. Briefly, 30 min after the final oral administration of treatment, ICR mice, with a lead block (7% of body weight) loaded on the tail, were individually placed into a glass cylinder, filled with water (25±1°C) to a depth of approximately 45 cm to prevent the mice from supporting themselves by touching the bottom with their feet. The total swimming time of mice was calculated from the moment they were placed in the water till completely exhausted, as evidenced by failing to rise to the water surface to breathe in a 10 s period, and drowning. The length of swimming time to exhaustion was evaluated as the degree of fatigue.

### Measurement of Hepatic Glycogen, Muscle Glycogen, and Hemoglobin

Thirty minutes after the final administration, the mice were sacrificed and blood samples were obtained from the inferior vena cava. Serum was obtained by centrifugation at 2,000×*g*, for 10 min at 37°C. Hemoglobin levels were determined using a hemoglobin detection kit (BestBio, China). The mice livers and leg muscles were immediately isolated, washed using physiological saline, and dried with absorbent paper. The hepatic glycogen and muscle glycogen levels were analyzed with commercially available detection kits (Solarbio, China) according to the manufacturer's instructions.

### Statistical Analysis

Data are expressed as the mean and standard error of the mean (mean±SEM). The significant difference was determined by one-way ANOVA, followed by Dunnett’s test for multi-group comparisons. All statistical analyses were performed using the SPSS 10.0 statistical analysis software. A *p*-value of < 0.05 was considered statistically significant. Additionally, *F* values with the degree of freedom (DF) were provided.

## Results

### Expression, Purification, and Enzyme Characterization of Recombinant β-xylosidases

**Fig. 2:**
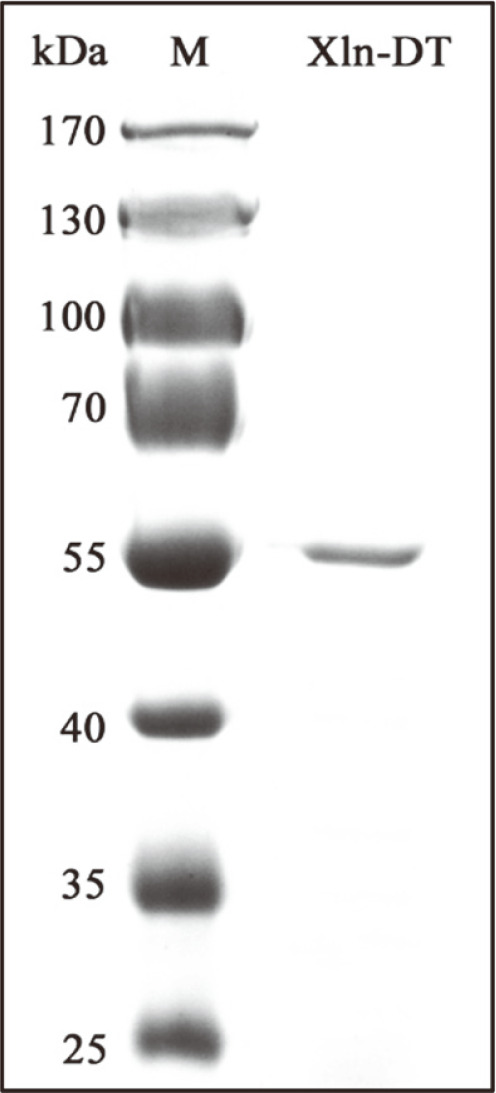
SDS-PAGE analysis of recombinant β-xylosidase Xln-DT expressed in *E. coli* BL21 (DE3) (Lane M, protein marker; Lane Xln-DT, purified β-xylosidase Xln-DT).

The GH39 β-xylosidase Xln-DT, derived from *D. thermophilum* DSM 3960, was heterologously expressed in *E. coli* BL21 (DE3) after incubation with 0.01 mM IPTG for 14-16 h. The crude enzyme solution was purified using a heat treatment at 75°C for 30 min, followed by Ni^2+^ affinity column chromatography. The purity of the protein sample was analyzed using 12% SDS-PAGE gel. As shown in Fig. 2, the target protein presented a single band with a molecular weight (MW) marginally greater than 55 kDa, without undesired bands, and related to the theoretical MW of the monomer (Fig. 2). The other four β-xylosidases were prepared in a similar manner (data were not shown). For the purified β-xylosidases Xln-DT, Tth xyB3, Tpxy3, XlnD, and Tth xyl, the enzyme activities based on the *p*NPX as substrate were 3.5 U/ml, 19.2 U/ml, 23.6 U/ml, 3.6 U/ml, and 7.0 U/ml, respectively. Therefore, the enzyme dosage for all R1 transformation experiments was based on the calculated enzyme activities when the *p*NPX as substrate.

With *p*NPX as the substrate, purified Xln-DT, Tth xyB3, Tpxy3, XlnD, and Tth xyl demonstrated apparent optimal temperatures of 75°C, 95°C, 90°C, 65°C, and 70°C, respectively, and apparent optimal pH values of 6.0, 6.0, 6.0, 4.5, and 6.5 (Table 1). Based on the results of enzymatic properties, we selected the optimal temperature and pH of each enzyme for R1 biotransformation.

### Optimization of Biotransformation

In this study, we investigated the specificity of five β-xylosidases, from different organisms and glycoside families, to bio-transform R1 to Rg1. Conventional β-xylosidases have been frequently used to hydrolyze the end bone of β-1, 4-linked xylopyranosyl; however, for R1, the 1, 2-glycosidic bond was targeted. As shown in Table 1, using 1 g/l of R1, enzyme dosage of 1 U/ml, and a reaction time of 1 h, under optimal pH and temperature conditions for each β-xylosidase, only GH39 β-xylosidase Xln-DT could comprehensively remove the outer xylose moiety at the C-6 carbon of R1, producing Rg1. This suggested that the high substrate specificity of β-xylosidases varies according to the enzyme structure and family sources.

**Fig. 3:**
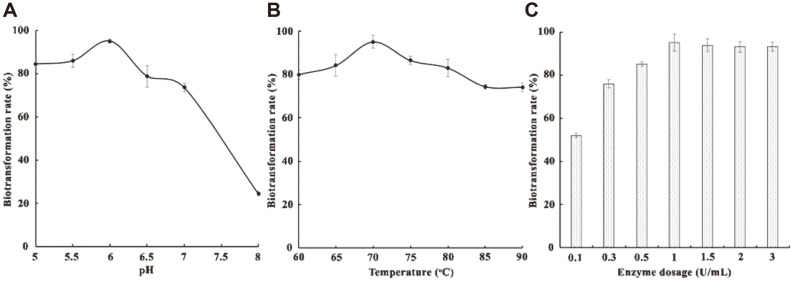
Optimization of biotransformation by recombinant β-xylosidase Xln-DT (A, the effect of pH on the biotransformation; B, the effect of temperature on the biotransformation; C, the effect of enzyme dosage on the biotransformation).

Therefore, for subsequent biotransformation experiments, the purified β-xylosidase Xln-DT from *D. thermophilum* was selected to optimize the biotransformation conditions (pH, temperature, and enzyme dosage) by single-factor experiments. For R1 biotransformation, the optimal pH was 6.0 (Fig. 3A), which was similar to the optimal pH of Xln-DT using *p*NPX as the substrate [29]. For R1 biotransformation, the optimal temperature using β-xylosidase Xln-DT is presented in Fig. 3B. In the temperature range of 60-80°C, the biotransformation rate was over 80%, and reached its highest point at 70°C. This may due to the excellent temperature stability of β-xylosidase Xln-DT in the range of 65-75°C. Due to the excellent pH and temperature stability of recombinant β-xylosidase Xln-DT, it has a good application prospect in the field of R1 biotransformation. The recombinant β-xylosidase Xln-DT demonstrated favorable factors, excellent for example, for potential applications in the field of R1 biotransformation. Furthermore, the longer half-life of the enzyme implies less enzyme consumption during practical applications. Under optimal temperature and pH (70°C, pH6.0) conditions, we investigated the enzyme dosage used for conversion. With increasing enzyme dosage, the biotransformation rate also improved, as shown in Fig. 3C. The optimal enzyme dosage of β-xylosidase Xln-DT was 1.0 U/ml. During a 30 min reaction, the biotransformation efficiency was found to exceed 95% for R1 at 70°C and pH 6.0, using recombinant β-xylosidase Xln-DT.

### Biotransformation of Notoginsenoside R1 to Ginsenoside Rg1

**Fig. 4:**
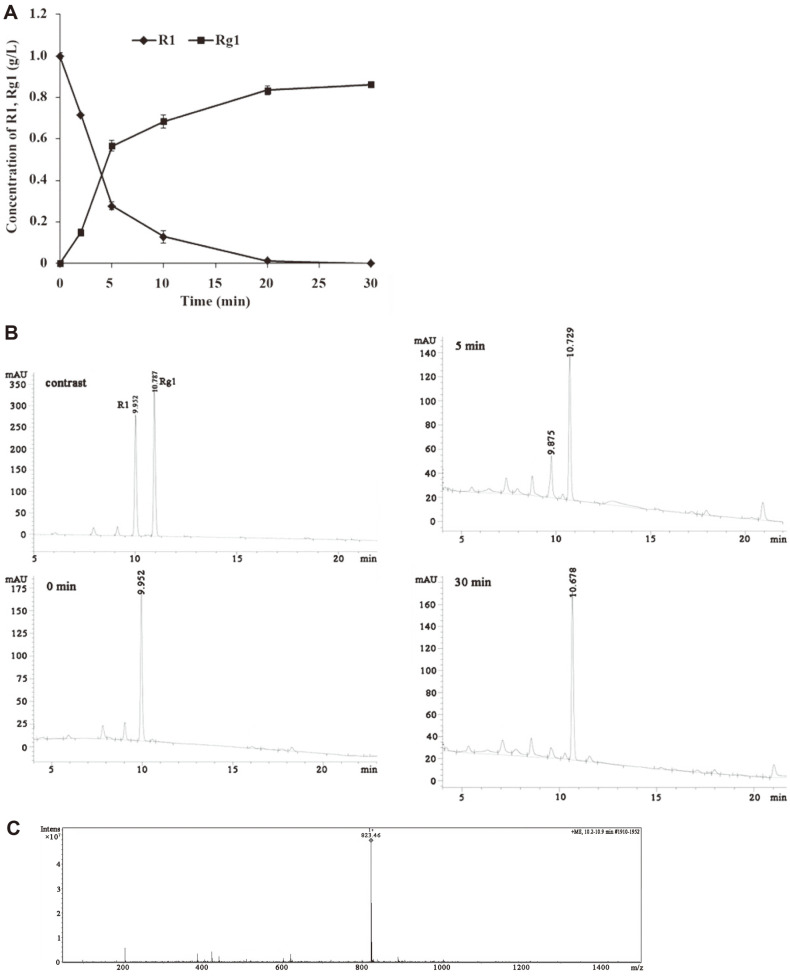
Liquid chromatography-mass spectrometry (LC-MS) and high-pressure liquid chromatography (HPLC) analyses of notoginsenoside R1 hydrolysis by recombinant β-xylosidase Xln-DT (A, a time-course analysis of the enzymatic action; B, HPLC analysis of the time-course of notoginsenoside R1 hydrolysis for 0, 5, and 30 min, respectively; C, LC-MS analysis of the product from notoginsenoside R1 hydrolysis).

To verify the biotransformation of R1 by Xln-DT, we performed a time-course analysis of the enzymatic action, and then analyzed the metabolite utilizing HPLC and LC-MS (Fig. 4). Hydrolysis of R1 yielded only a single distinct metabolite. As shown in Fig. 4A, the reaction mixture, containing 50 mM buffer (pH 6.0), 1 g/l of R1, and 1 U/ml of Xln-DT, was incubated for 30 min at 70°C. R1 was almost completely converted after 20 min; however, the transformation times using β-xylosidases from *T. thermosaccharolyticum* [21] and *Sphingomonas* sp. JB13 [22] were 4 h and 24 h, respectively, indicating that β-xylosidase Xln-DT, derived from *D. thermophilum*, substantially increased the conversion efficiency. In total, 1 g/l of R1 was transformed into 0.86 g/l of Rg1 after a reaction time of 30 min, with a corresponding molar conversion yield of 100%. LC-MS was used to determine structural information regarding the metabolite converted from R1. Based on the LC-MS spectrum (Fig. 4C), only one metabolite (*m/z*: 823.46, [M+Na]^-^) was detected following the loss of a xylose residue. Ginsenoside Rg1 was confirmed as the single hydrolysis product. This indicated that Xln-DT could selectively cleave the β-1, 2-glycosidic linkage of R1, without attacking the other glycosidic linkages. To date, the thermotolerant β-xylosidase Xln-DT from the GH39 family is one of the few enzymes known to hydrolyze R1 to Rg1, without other byproducts. Notably, the enzymatic hydrolysis time is short and the bioconversion efficiency is high, suggesting that this recombinant Xln-DT has great potential for industrial applications, especially in the biotransformation and production of natural medicinal agents.

**Fig. 5:**
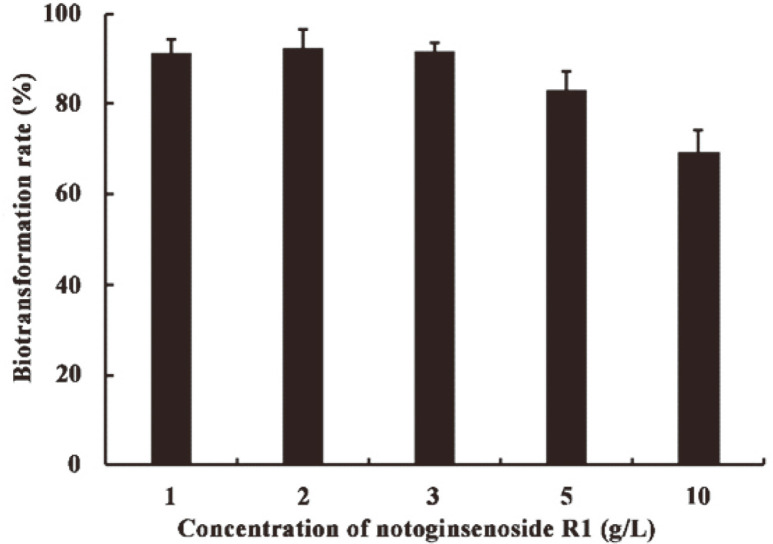
The influence of notoginsenoside R1 concentration on the biotransformation rate.

Additionally, the substrate concentration was a limiting factor affecting bioconversion efficiency. To examine the effect of R1 biotransformation induced by Xln-DT, different R1 concentrations (from 1 g/l to 10 g/l) were incubated with Xln-DT (1 U/ml), at pH 6.0 and 70°C, for 30 min (Fig. 5). Compared with concentrations of 1 g/l and 10 g/l, the activity of Xln-DT was marginally inhibited with a high concentration of R1. When the R1 concentration was below 3 g/l, the biotransformation rate was approximately 100%. On increasing the R1 concentration to 10 g/l, the bioconversion rate decreased to 70%. Moreover, β-xylosidase Xln-DT presented a *K_i_* for xylose above 3 M, indicating the potential advantages of this enzyme in industrial applications as xylose is a strong inhibitor of β-xylosidase.

Under optimal conditions, 300 mg of R1 was biotransformed by purified β-xylosidase Xln-DT. The final content in the sample was determined as 192 mg, with a yield of 74.4%.

### Effects of R1 and Rg1 on Body Weight Changes

The changes in the body weight of ICR mice during the experimental period are shown in Table 2. The body weight was recorded before the experiment (initial), after 7 days (intermediate), and after 14 days (final). No significant difference in the body weight of ICR mice was observed in the R1 and Rg1 groups when compared with the negative control during initial, intermediate, and final stages of the experiment (*F*=1.08, *p*=0.7451, 0.3737, 0.7712, 0.1974 (*p* > 0.05) for R1 low dosage, R1 high dosage, Rg1 low dosage and Rg1 high dosage, respectively). This result suggested that R1 and Rg1 induced no toxic effect in mice and did not inhibit the growth of mice.

### Evaluation of Anti-Fatigue Activity on Mice

**Fig. 6:**
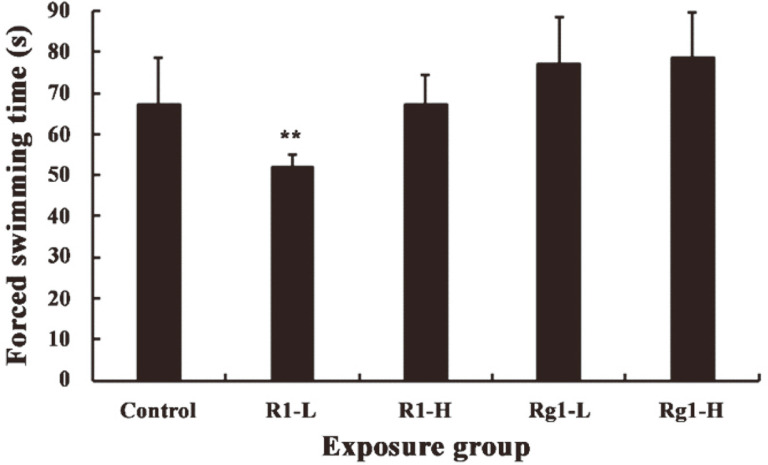
Effect of notoginsenoside R1 and ginsenoside Rg1 on forced swimming test in ICR mice (Data are mean ± SE. Compared with control, **p < 0.05. PBS buffer was administered to rats as a negative control, 9 ICR mice were observed and tested for each group during the experimental period).

In this study, the anti-fatigue activity of R1 and Rg1 was evaluated by determining the weight-loaded swimming endurance capacity. In rodents, the forced swimming test is a suitable experimental model frequently used in previous investigations [31, 32]. The length of swimming time indicates the degree of fatigue in animal movement. As expected, compared with the negative control group, the swimming time of the two Rg1 groups (5 and 20 mg/kg, respectively) increased by 15.3% and 17.3%, respectively, suggesting that the swimming time in mice was prolonged by Rg1 in the low-dose and high-dose groups. Moreover, the high concentration Rg1 group demonstrated more prominent effects. However, R1 failed to significantly promote weight-loaded swimming time in mice, in both the low and high concentration groups (Fig. 6). Furthermore, in the low concentration R1 group, the forced swimming time was significantly reduced (*p* < 0.05). However, the index of forced swimming time alone cannot be used to comprehensively evaluate the anti-fatigue activity of Rg1 and R1.

**Fig. 7:**
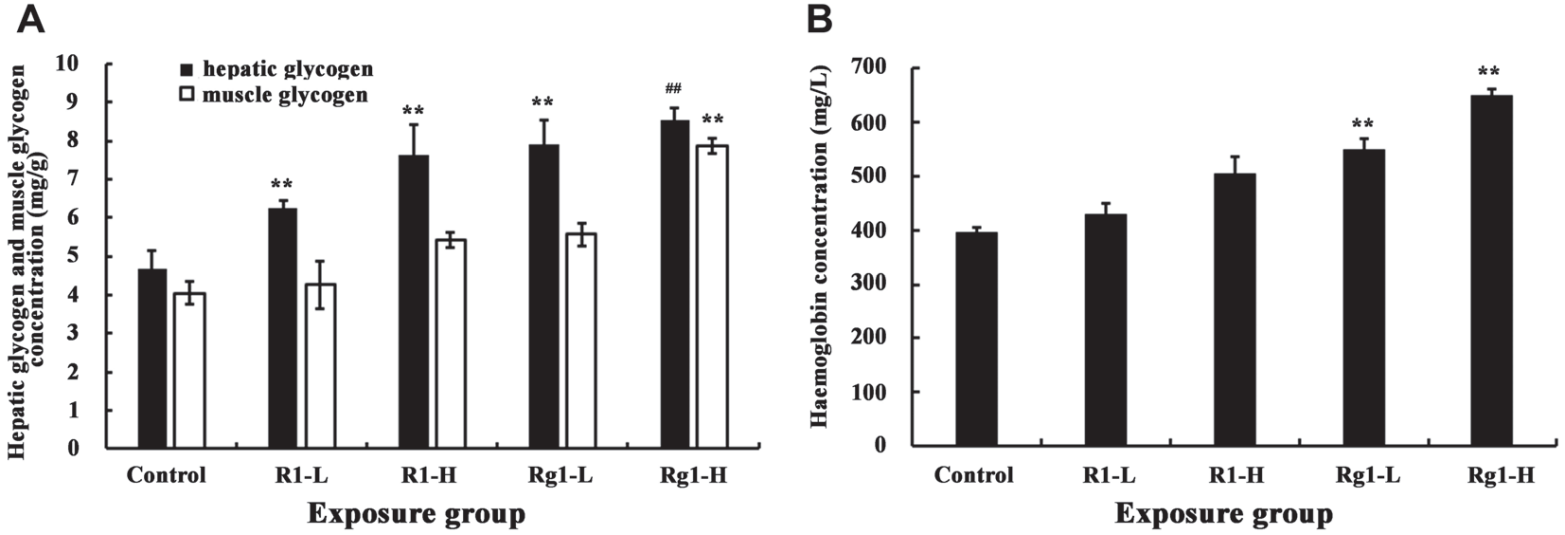
Effect of notoginsenoside R1 and ginsenoside Rg1 on hepatic glycogen, muscle glycogen, and hemoglobin levels in ICR mice (Data are mean ± SE. Compared with control, **p < 0.05, ## *p* < 0.01. A, hepatic glycogen and muscle glycogen levels in ICR mice, black square: hepatic glycogen, white square: muscle glycogen; B, hemoglobin levels in ICR mice).

Additionally, hepatic glycogen, muscle glycogen, and hemoglobin (Hb) are representative blood biochemical parameters related to fatigue [33]. The liver is an important tissue for energy conservation and utilization, converting lactate back into glycogen, and releasing glycogen into the blood [34]. Glycogen is the main source of energy for muscle activity. Therefore, the glycogen content can indicate the speed and degree of fatigue. If hepatic and muscle glycogen in the test group is significantly higher than that of the control group, and the difference is significant, indicating that the test group can provide more energy for utilization by the body, by increasing the hepatic and muscle glycogen reserve to achieve anti-fatigue effects [35, 36]. As shown in Fig. 7A, exposure to the forced swimming test resulted in an increase in hepatic and muscle glycogen in the test group (R1 and Rg1). In the Rg1 groups (5 and 20 mg/kg), hepatic and muscle glycogen levels were significantly increased by 69.9-83.3% and 36.9-93.6% (*p* < 0.05), respectively, when compared with those observed in the negative control group. Moreover, the anti-fatigue activity was positively correlated with the Rg1 content; the higher the concentration, the more marked the anti-fatigue effect. Therefore, the increased levels of hepatic and muscle glycogen may explain one pathway of the anti-fatigue effects induced by Rg1. Similarly, in the R1 groups (5 and 20 mg/kg), the concentration of hepatic and muscle glycogen was higher than that in the control group but lower than that observed in the Rg1 groups, indicating that R1 could also demonstrate anti-fatigue activity in mice. Furthermore, these results confirmed the anti-fatigue activities of saponins from natural products, such as *Panax ginseng* and *Astragalus membranaceus* [36, 37]. Furthermore, after 14 days of treatment, hemoglobin levels of the Rg1 groups were significantly higher than those observed in the negative group, especially in the high-dose group (Fig. 7B). However, no significant differences were observed in hemoglobin levels between the negative control and the R1 groups (*p* > 0.05). Overall, these findings revealed that Rg1 can increase the anti-fatigue activity by prolonging the weight-load swimming time, significantly increasing the hepatic glycogen and muscle glycogen, and enhancing the hemoglobin index.

## Discussion

To date, a large number of investigations have reported that glycosidases, including β-glucosidases, α-rhamnosidases, and β-xylosidases, are capable of transforming natural active substances such as flavonoids, astragalosides, terpenoids, and ginsenosides [38-40]. Distinct glycosidases derived from various sources and families are known to differ in their protein structure and biotransformation specificity toward certain natural active materials. For instance, only two β-D-xylosidases belonging to GH3, from *Lentinula edodes* and *Dictyoglomus turgidum*, reportedly remove xylosyl groups from 7-β-xylosyl-10-deacetyltaxol [41, 42].

Owing to the lack of specificity, as well as the high conversion rate during enzymatic hydrolysis of R1, the biological enzymes investigated in this study demonstrated relative specificity, with some indicating absolute specificity. For natural active substances with different skeletons, the types of bonds (such as a β-1, 2-glycoside bond, β-1, 4-glycoside bond) and positions of xylose linkage differ, including the specificity and catalytic efficiency of β-xylosidase. In this study, recombinant Xln-DT, belonging to the GH39 family, demonstrated a sequence homology of less than 30% with the other three β-xylosidases of GH3 family, derived from *Thermotoga thermarum* DSM 5069 [23], *T. petrophila* DSM 13995 [25], and *Aspergillus niger* NL-1. Furthermore, it exhibits a 32.1% identity with GH120 β-xylosidase from *T. thermosaccharolyticum* DSM 571, sharing 71.7% and 28.0%identities with GH39 β-xylosidases from *T. thermosaccharolyticum* (GenBank Accession No. YP_003851597.1)[21] and *Sphingomonas* sp. JB13 (GenBank Accession No. AZC12019.1) [22], respectively. Different β-xylosidases have different enzymatic properties due to their different sources, glycoside hydrolase families and structures. As shown in Table 1, only GH39 β-xylosidases (Xln-DT and two reported β-xylosidases) cleaved the outer xylose moiety of R1 at the C-6 position. Although the three β-xylosidases belong to the same family, they are derived from different sources, resulting in different biotransformation efficiencies. The GH39 β-xylosidase from *T. thermosaccharolyticum* can completely hydrolyze R1 to Rg1 at 60°C, pH 6.5, and 10.2 U/ml enzyme dosage for 4 h [21]. The GH39 β-xylosidase rJB13GH39 from *Sphingomonas* sp. JB13 can hydrolyze R1 to produce Rg1 at 30°C and pH 4.5 for 24 h, demonstrating a molar conversion of 90.3% [22]. However, Xln-DT was able to completely hydrolyze R1 to Rg1 at 70°C, pH 6.0, and 1 U/ml of the enzyme in 30 min, indicating that Xln-DT demonstrates a strong affinity and high catalytic efficiency for R1.

Owing to the discovery of some thermophilic bacteria and fungi, some β-xylosidases with high optimal temperature and heat resistance have been reported in succession. For enzymes, temperature stability is an important index to assess the catalytic performance. During biological catalysis and transformation of natural active substances, β-xylosidases with good thermal stability possess the following advantages: (i) if the solubility of the natural active substance is poor, its solubility will increase with an increase in temperature, which allows the reaction rate of the enzyme to be increased, and enzyme amount to be decreased; (ii) high temperature can reduce the probability of bacterial infection in the biotransformation system; (iii) an enzyme with good thermal stability has a longer storage time and is not easily inactivated, which can greatly reduce the cost of enzyme production. Accordingly, the high-temperature resistance of Xln-DT may contribute to its high conversion efficiency for notoginsenoside R1, which will be more conducive for cost reduction during industrial production processes.

β-Xylosidase demonstrates important application value in the field of industrial biotechnology. However, if the concentration of the hydrolysis product xylose is markedly high, it will feedback inhibit the β-xylosidase activity, limiting its practical applications. Notably, the xylose tolerance of the reported β-xylosidases ranges between 1.7 and 1,000 mM. For example, Kirikyali has cloned and heterologously expressed a β-xylosidase from *Neurospora crassa* with a *K*_i_ value of 1.72 mM [43]; Yang has cloned two GH43 family β-xylosidases from *Humicola inosoles*, with *K*_i_ values of 79 and 292 mM [44]; Shi has cloned a bifunctional enzyme of β-xylosidase/α-arabinoside from *T. thermarum* with a *K*_i_ value of 1000 mM [23]. According to our previous study, Xln-DT demonstrates an extremely high xylose resistance, presenting a xylose resistance coefficient *K*_i_ value of 3300 mM, which remains the highest xylose tolerance coefficient reported. Hence, when the concentration of R1 was increased to 10 g/l, the biotransformation rate was more than 70% within 30 min, indicating that Xln-DT would not be inactivated owing to the feedback inhibition of xylose, which further demonstrated that Xln-DT possesses potential advantages in industrial applications.

Fatigue is a physiological reaction, which has been categorized as a deficiency of labor and other diseases in traditional Chinese medicine [45]. Long-term fatigue can easily lead to various diseases, including aging, hypotension, cancer, liver dysfunction, depression and HIV infection [46, 47]. However, only a few targeted pharmacological drugs can treat fatigue [48]. In recent years, some natural food ingredients and active substances from forest plants have revealed beneficial effects in fatigue. Hu has reported that purified purple passion fruit epicarp anthocyanins could relieve fatigue symptoms [49]. Kim has evaluated the anti-fatigue effect of *Prunus mume* vinegar in high-intensity exercised rats, suggesting that *P. mume* vinegar could be used as a functional material against fatigue [50]. Natural products derived from forest plants such as *ginseng*, *Panax notoginseng*, and *Astragalus membranaceus*, have several pharmaceutical benefits, including anti-cancer effects, osteogenesis, and stimulation of immune functions [51-53]. In Asian countries, including China and South Korea, one traditional use of ginseng is an anti-fatigue agent [36, 54]. Yang has reported the effects of ginsenoside Rg3 on fatigue resistance, revealing that ginsenoside Rg3 could improve exercise ability, as well as anti-fatigue effects, by increasing SIRT1 deacetylase activity [55]. However, limited reports are available on the anti-fatigue activity and activity comparison between other ginsenoside monomers. This study first demonstrated and compared the anti-fatigue activity between R1 and Rg1, by evaluating hepatic glycogen, muscle glycogen, and hemoglobin accumulation in vivo. After 14 days of treatment, Rg1 was able to prolong the forced swimming time in mice. Furthermore, in treated Rg1 groups, hemoglobin levels were significantly higher than that those in negative and R1 groups, suggesting that Rg1 can increase the anti-fatigue activity by significantly increasing the hepatic glycogen and muscle glycogen, as well as elevating the hemoglobin index. Collectively, these findings suggest that the enzymatic transformation of R1 could significantly improve the purity and yield of Rg1, and Rg1 could be used as a functional material to resist fatigue.

## Figures and Tables

**Table 1:** The biotransformation rate of notoginsenoside R1 by different β-xylosidases.

β-xylosidase	Source	Sequence homology	GH family	Optimum temperature^a^	Optimum pH^b^	Notoginsenoside R1 biotransformation rate (%)	Ref.
Xln-DT	*Dictyoglomus thermophilum* DSM 3960	100^c^	39	75°C	6.0	100	[29]
Tth XynB3	*Thermotoga thermarum* DSM 5069	27.3	3	95°C	6.0	0	[23]
Tpxy3	*Thermotoga petrophila* DSM 13995	26.3	3	90°C	6.0	0	[25]
XlnD	*Aspergillus niger* NL-1	25.4	3	65°C	4.0	0	This study
Tth Xyl	*Thermoanaerobacterium thermosaccharolyticum* DSM 571	32.1	120	70 °C	6.5	0	This study
Tth39	*Thermoanaerobacterium thermosaccharolyticum*	71.7	39	60 °C	6.5	100	[21]
JB13GH39	*Sphingomonas* sp. JB13	28.0	39	50 °C	4.5	90	[22]

^a^The determination of optimum temperature for β-xylosidases was using *p*-Nitrophenyl-β-D-xylopyranoside as the substrate.

^b^The determination of optimum pH for β-xylosidases was using *p*-Nitrophenyl-β-D-xylopyranoside as the substrate.

**Table 2:** Effects of notoginsenoside R1 and ginsenoside Rg1 on body weight in ICR mice.

Group	Treatment (mg/kg)	Body weight (g)^a^		

		Initial	Intermediate	Final
Control^b^	-	27.41 ± 1.30	31.00 ± 2.09	35.42 ± 2.81
R1-low dosage	5	27.63 ± 1.28	30.41 ± 3.91	33.82 ± 3.50
R1-high dosage	20	27.78 ± 0.70	31.60 ± 0.74	34.92 ± 0.69
Rg1-low dosage	5	27.41 ± 1.02	30.67 ± 2.27	33.88 ± 2.35
Rg1-high dosage	20	28.27 ± 0.92	32.23 ± 2.11	35.11 ± 1.60

^a^Data are expressed as mean ± S.E.;

^b^phosphate buffer saline was given to mice as a negative control; 12 ICR strain mice were observed and tested for each group during the experimental period.
